# Costunolide, a Sesquiterpene Lactone, Protects Against Platelet Activation and Thrombus Formation

**DOI:** 10.3390/cells15100938

**Published:** 2026-05-20

**Authors:** Joen-Rong Sheu, Kuan-Hung Lin, Ray-Jade Chen, Hao-Ping Chia, Ting-Yu Chen, Thanasekaran Jayakumar, Hsueh-Hsiao Wang, Hsien-Yu Peng, Jiun-Yi Li, Wan-Jung Lu

**Affiliations:** 1 Graduate Institute of Medical Sciences, College of Medicine, Taipei Medical University, Taipei 110, Taiwan; sheujr@tmu.edu.tw; 2 Department of Pharmacology, School of Medicine, College of Medicine, Taipei Medical University, Taipei 110, Taiwan; b101107142@tmu.edu.tw; 3 Institute of Biomedical Sciences, College of Medicine, MacKay Medical University, New Taipei City 252, Taiwan; linkh@mmu.edu.tw (K.-H.L.); tingyu851126@gmail.com (T.-Y.C.); 4 Department of Surgery, School of Medicine, College of Medicine, Taipei Medical University, Taipei 110, Taiwan; rayjchen@tmu.edu.tw; 5 Division of General Surgery, Department of Surgery, Taipei Medical University Hospital, Taipei 110, Taiwan; 6 Department of Ecology and Environmental Sciences, Pondicherry University, Puducherry 605014, India; tjayakumar@pondiuni.ac.in; 7 School of Medicine, College of Medicine, MacKay Medical University, New Taipei City 252, Taiwan; okul.wang@gmail.com (H.-H.W.); hsien.yu@gmail.com (H.-Y.P.); 8 Department of Surgery, MacKay Memorial Hospital, Taipei 104, Taiwan; 9 Department of Optometry, College of Medicine, MacKay Medical University, New Taipei City 252, Taiwan; 10 Traditional Herbal Medicine Research Center, Taipei Medical University Hospital, Taipei 110, Taiwan

**Keywords:** cardiovascular diseases, costunolide, GPVI, platelet activation, thrombus formation

## Abstract

**Highlights:**

**What are the main findings?**

- Costunolide exerts antiplatelet and antithrombotic activities without bleeding tendency.
- Costunolide inhibits platelet activation through suppressing the PLCγ2-PKC, Akt, and MAPK signaling pathways.

**What are the implications of the main findings?**

- Costunolide may serve as a lead compound for the synthesis and discovery of novel and potent antiplatelet agents.
- Costunolide may provide a therapeutic strategy for patients with CVD, particularly stroke and heart attack.

**Abstract:**

**Background/Objectives**: Circulating platelets mediate physiological hemostasis and are implicated in pathological thrombosis, which can cause vascular occlusion, leading to heart attacks or strokes. Costunolide is a sesquiterpene lactone extracted from *Saussurea lappa*. Although this lactone has multiple biological effects, including anti-inflammatory and antioxidant effects, that help slow the progression of atherosclerosis, its influence on platelet activation remains unclear. In this study, we examined the potential antiplatelet and antithrombotic effects of costunolide. **Methods**: We used platelet aggregation, flow cytometry, and Western blot analysis to examine its in vitro antiplatelet effects. **Results**: Our results indicated that costunolide inhibited platelet aggregation induced by collagen, but not by thrombin or the thromboxane A_2_ analog U46619, suggesting that costunolide selectively inhibits collagen-induced platelet activation. Additionally, costunolide blocked collagen-mediated granule release, calcium mobilization, and glycoprotein IIb/IIIa (GPIIb/IIIa) activation. Costunolide also inhibited phospholipase Cγ2 (PLCγ2), pleckstrin (a downstream target of protein kinase C), Akt, and mitogen-activated protein kinase. Moreover, it prevented collagen/epinephrine-induced pulmonary thrombosis and increased the survival rate of mice. Furthermore, costunolide delayed thrombus formation in the mesenteric vessels while it did not significantly affect hemostasis, suggesting it exhibits antithrombotic activity without bleeding tendency. These findings indicate that costunolide can block PLCγ2-PKC, Akt, and MAPK signaling pathways and subsequent granule release, calcium mobilization, and GPIIb/IIIa activation, eventually impeding platelet activation, platelet aggregation, and thrombus formation. **Conclusions**: In conclusion, besides its multiple biological activities that are beneficial for slowing the progression of atherosclerosis, we also demonstrated the antiplatelet and antithrombotic activities of costunolide. These effects highlight the therapeutic potential of costunolide in the treatment of patients with cardiovascular disease, particularly stroke and heart attack.

## 1. Introduction

Cardiovascular disease (CVD) is the leading cause of death worldwide. In 2022, an estimated 19.8 million people died from CVD, accounting for approximately 32% of all global deaths. Of these deaths, 85% were attributable to heart attacks and strokes [1]. These statistics indicate that CVD, particularly that manifesting in heart attacks and strokes, remains a major threat to global health. Atherosclerosis, a major cause of heart attack and stroke, is a chronic inflammatory disorder characterized by endothelial cell activation, immune cell recruitment, and smooth muscle cell proliferation. Platelets have been reported to participate in the initiation and progression of atherosclerosis [2,3]. In ApoE knockout mice, platelet adhesion to the carotid endothelium was found to precede the manifestation of atherosclerotic lesions, implicating a role of platelets in the early stage of the atherogenetic process [4]. Atherosclerosis is also exacerbated by activated wild-type platelets, but not P-selectin–deficient ones, by facilitating monocyte recruitment to the lesion surface, suggesting that platelet-derived P-selectin serves as a vital mediator in enhancing atherosclerotic development [5,6]. Indeed, platelets reportedly have atherogenic effects by inducing monocyte migration and recruitment into atherosclerotic plaques and then skewing macrophages to a pro-inflammatory phenotype [7]. Therefore, mitigating platelet activation may be an effective strategy for slowing the progression of atherosclerosis and preventing heart attacks and strokes. However, the antiplatelet drugs used in clinical settings are often associated with a bleeding tendency, which limits their use. Therefore, novel antiplatelet agents with a reduced bleeding risk are urgently required.

Platelets play a pivotal role in maintaining hemostasis. Upon vascular injury, circulating platelets adhere and become activated through interactions with exposed extracellular matrix proteins, including von Willebrand factor and collagen. Collagen binds to the collagen receptor glycoprotein VI (GPVI), which initiates GPVI-mediated signaling involving Src family kinases (Fyn and Lyn), phospholipase Cγ2 (PLCγ2), and protein kinase C (PKC). This signaling cascade ultimately triggers granule release and platelet activation, which amplifies platelet responses and helps recruit additional circulating platelets to promote platelet plug formation and prevent blood loss [8,9]. Under pathological conditions such as atherosclerosis, platelets may become hyperreactive and cause vessel occlusion through thrombus formation.

**Figure 1:**
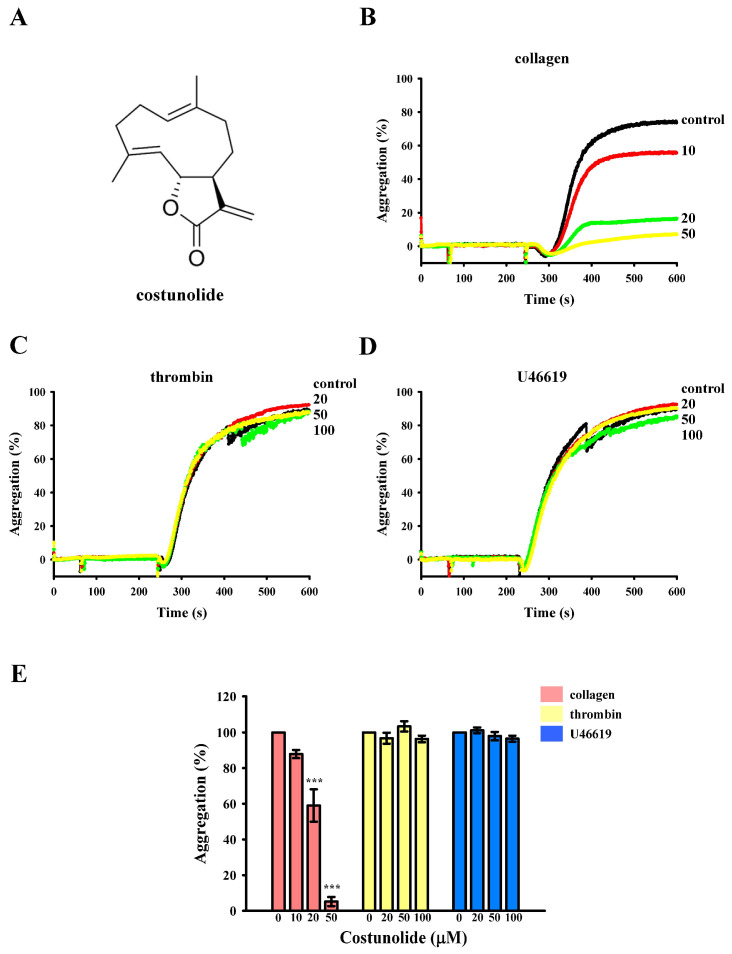
Costunolide selectively inhibits collagen-induced platelet aggregation. (**A**) Structure of costunolide. Washed platelets (3.6 × 10^8^ cells/mL) were pretreated with 0.1% DMSO (solvent control) or costunolide (10–100 µM) prior to the addition of (**B**) 1 μg/mL collagen, (**C**) 0.02 U/mL thrombin, and (**D**) 1 µM U46619 to trigger platelet aggregation. (**E**) Statistical analysis of the platelet aggregation results presented in panels (**B**–**D**). The results presented in panels (**B**–**D**) are representative of four similar experiments. Data (**E**) are presented as means ± standard errors of the mean (*n* = 4). *** *p* < 0.001, compared with the DMSO (solvent control) group.

Costunolide is a sesquiterpene lactone (Figure 1A) extracted from *Saussurea lappa*. It possesses multiple biological effects, including anti-inflammatory and anticancer effects. For example, it was reported to normalize neuroinflammation by inhibiting the microglial Akt/mTOR/NF-κB pathway in a mouse model of depression [10]. It was also demonstrated to ameliorate chronic atrophic gastritis by activating Nrf2 [11] and to reduce obesity- and hyperglycemia-induced cardiomyopathy by activating Nrf2 and downregulating NF-κB [12,13]. In addition, costunolide was reported to mitigate ischemia-induced brain damage by inhibiting CaMKII phosphorylation [14] and to alleviate atherosclerosis by inactivating NF-κB in ApoE-knockout mice [15]. In cancer, it inhibits colorectal cancer cell growth by arresting the cell cycle and inducing apoptosis by targeting Akt [16]. It also inhibits osteosarcoma cell growth and metastasis by suppressing the STAT3 signaling pathway [17].

Despite its advantages, whether costunolide can prevent platelet activation and thrombus formation remains unclear. Therefore, the present study investigated the mechanisms underlying its potential antiplatelet and antithrombotic effects.

## 2. Materials and Methods

### 2.1. Materials

Costunolide (purity ≥ 98%) was supplied by Cayman Chemical (Ann Arbor, MI, USA). Collagen, thrombin, and the thromboxane A_2_ analog U46619 were supplied by Chrono-Log (Havertown, PA, USA). Alexa Fluor 647 fibrinogen conjugate and Fura 2-AM (F1221) were sourced from Thermo Fisher Scientific (Waltham, MA, USA) (including Molecular Probes, Eugene, OR, USA). Phycoerythrin-conjugated anti-P-selectin antibodies were ordered from BioLegend (San Diego, CA, USA). Cell Signaling Technology (Beverly, MA, USA) provided monoclonal antibodies targeting Akt (2920S) and ERK1/2 (extracellular signal-regulated kinase 1/2; 9107S), alongside polyclonal antibodies specific for PLCγ2 (3872S), c-Jun N-terminal kinase (9252S), p38 MAPK (9212S), phosphorylated (Ser^473^) Akt (9271S), phosphorylated (Ser) PKC substrate (pleckstrin, p47; 2261S), phosphorylated (Ser^180^/Tyr^182^) p38 MAPK (9211S), and phosphorylated (Thr^183^/Tyr^185^) JNK (9251S). Anti-phosphorylated (Tyr^753^) PLCγ2 (ab133455) was supplied by Abcam (Cambridge, UK). GeneTex (Irvine, CA, USA) was the source for total p47 (GTX17020) and phosphorylated ERK1 (Thr^202^/Tyr^204^)/ERK2 (Thr^185^/Tyr^187^) (GTX82696) polyclonal antibodies. Horseradish peroxidase (HRP)-conjugated secondary antibodies, including AffiniPure goat anti-rabbit (111-035-003), goat anti-mouse (115-035-003), and donkey anti-goat (705-035-003) IgG, were supplied by Jackson ImmunoResearch Laboratories (West Grove, PA, USA). A SuperLight Chemiluminescent HRP kit and Hybond P polyvinylidene difluoride membranes were purchased from Cytiva (Marlborough, MA, USA). Costunolide was dissolved in DMSO and stored at 4 °C until use.

### 2.2. Platelet Aggregation Assay

In compliance with the Declaration of Helsinki, the study protocol received approval from the Joint Institutional Review Board of Taipei Medical University, Taipei, Taiwan (TMU-JIRB-No. N202202001). Healthy volunteers provided the blood samples used in this investigation. Before donating blood samples, healthy volunteers abstained from any medication for two weeks. These samples were immediately processed to prepare platelet suspensions. All volunteers provided written informed consent. A total of 24 participants aged 21–40 years were recruited (13 males and 11 females). Following a previously established protocol [18], preparation of human platelet suspensions was conducted. First, we mixed blood with acid citrate dextrose (9:1, *v*/*v*) and performed a 10 min centrifugation at 120× *g* to yield platelet-rich plasma. The collected supernatant was added with MilliporeSigma (Burlington, MA, USA) prostaglandin E_1_ and heparin, and spun at 500× *g* for 10 min. The pellet was washed twice and suspended in Tyrode’s solution (3.5 mg/mL bovine serum albumin from Bionovas Biotechnology, Toronoto, ON, Canada, 1 mM Ca^2+^).

A turbidimetric AggRAM system (Helena Laboratories, Beaumont, TX, USA) was utilized to determine platelet aggregation as previously reported [18]. Platelet suspensions (3.6 × 10^8^ cells/mL) were incubated with costunolide (10–100 µM) or 0.1% DMSO (solvent control) for 3 min. Aggregations induced by 1 µg/mL collagen, 0.02 U/mL thrombin, or 1 µM U46619 were then evaluated over a 6 min reaction window.

### 2.3. Western Blot Analysis

Washed platelets (3.6 × 10^8^ cells/mL) underwent a 3 min pretreatment with costunolide (20 and 50 µM) or 0.1% DMSO, followed by activation with 1 µg/mL collagen. After terminating the reaction, the platelets were lysed with 200 μL of lysis buffer for 1 h at −20 °C. Supernatants collected after centrifugation (5000× *g*, 5 min) were quantified and 80 μg of protein were separated using 12% SDS-PAGE. Proteins were transferred to PVDF membranes (Molecular Probes, Thermo Fisher Scientific) through a semidry method. After blocking with 5% BSA in TBST (100 mM NaCl, 10 mM Tris-base, and 0.01% Tween-20) at 25 °C, the membrane was washed and probed with various primary antibodies (1:1000) for 2 h. After thrice washing, horseradish peroxidase-linked secondary antibodies (1:3000) were applied for 1 h at 25 °C. Finally, immunoreactive signals were detected using an enhanced chemiluminescence system and analyzed on a Celvin S system (Biostep GmbH, Burkhardtsdorf, Germany).

### 2.4. Flow Cytometry

Flow cytometric analysis followed an established method [19]. Platelet suspensions (3.6 × 10^8^ cells/mL) underwent a 3 min pretreatment with costunolide (20 and 50 µM) or 0.1% DMSO. Activation was carried out at 37 °C in glass cuvettes using 1 µg/mL collagen. The resulting activated platelets were fixed and then stained for 30 min at 37 °C with Alexa Fluor 647-fibrinogen conjugate and phycoerythrin–anti-P-selectin antibodies. Sample volumes of 1 mL were immediately acquired on a CytoFLEX flow cytometer (Beckman Coulter, Brea, CA, USA), capturing 10,000 platelet events per group. Data are expressed as fold changes in mean fluorescence intensity relative to the resting group. Statistical significance was assessed by comparing the costunolide-treated group against the positive control (DMSO + collagen) group to evaluate inhibitory efficacy. All findings were verified through at least four independent replicates.

### 2.5. Adenosine Triphosphate Release and Calcium Mobilization Assay

Washed platelets (3.6 × 10^8^ cells/mL) pretreated with luciferase/luciferin and Fura 2-AM were treated with costunolide (20 and 50 µM) or DMSO (0.1%, solvent control) for 3 min at 37 °C. Platelets were then activated with 1 µg/mL collagen. Luminescence (ATP) and fluorescence signals (calcium) were recorded on a Hitachi F-7000 Fluorescence Spectrometer (Hitachi, Tokyo, Japan).

### 2.6. Animals

Male ICR mice (weight, 20–25 g; age, 5–6 weeks) were supplied by BioLASCO Taiwan (Taipei city, Taiwan). Animals were maintained in a temperature-controlled environment (22 ± 2 °C) under a 12 h light/dark cycle with ad libitum access to chow and water. Animals were monitored daily for signs of distress. Animals were randomly assigned to experimental groups. All animal procedures adhered to the Guide for the Care and Use of Laboratory Animals [20] and received formal approval from the Institutional Animal Care and Use Committee (IACUC) of Taipei Medical University (LAC-2022-0028). Humane endpoints were established according to institutional guidelines. The in vivo dose of costunolide was calculated using a body surface area-based translation method [21] (human equivalent dose (mg/kg) = mouse dose (mg/kg) × (mouse *K_m_*/human *K_m_*)), applying *K_m_* factors of 3 for mice and 37 for humans. Upon study completion, the mice were humanely euthanized via gradual carbon dioxide displacement (30% chamber volume per minute). Death was verified by checking for the permanent absence of both cardiac and respiratory activities.

### 2.7. Pulmonary Embolism in Mice

Murine pulmonary thromboembolism was provoked in a mouse model via simultaneous administration of collagen and epinephrine, following an established method [19]. Briefly, anesthetized mice (*n* = 6 per group) were intravenously administered either 1.1 g/kg DMSO, costunolide (4.6 and 11.4 mg/kg), or 20 mg/kg aspirin. Next, a mixture of 0.6 mg/kg collagen and 0.2 mg/kg epinephrine (MilliporeSigma) was delivered by the tail vein to trigger lung thrombosis. After the injection of collagen/epinephrine, mice were removed from anesthesia and monitored for 24 h. Upon experiment completion, excised lung tissues were harvested and subjected to hematoxylin and eosin staining for subsequent histological imaging. Surviving mice were humanely sacrificed at the 24 h endpoint.

### 2.8. Tail-Bleeding Time

Tail bleeding was induced according to a previously described method [19]. Briefly, anesthetized mice received an intravenous injection of 1.1 g/kg DMSO, costunolide (4.6 and 11.4 mg/kg), or 20 mg/kg aspirin 10 min before injury. Each experimental group consisted of six animals (*n* = 6). To initiate bleeding, a tail transection was performed 3 mm from the tip, followed by immediate immersion in saline. The bleeding duration was monitored until it ceased for at least 10 s, with a maximum observation period of 10 min.

### 2.9. Statistical Analysis

All statistical analyses were conducted using SigmaStat (version 3.5; SYSTAT Software). Data are expressed as means ± standard errors of the mean (n represents the sample size per experimental group within each independent assay). Statistical variances between multiple groups were verified through one-way ANOVA paired with the Newman–Keuls post hoc test. Survival profiles were plotted as Kaplan–Meier curves and evaluated with the log-rank test. Statistical significance was defined as a *p*-value < 0.05.

## 3. Results

### 3.1. Costunolide Selectively Inhibits Collagen-Induced Platelet Aggregation

In this study, three platelet inducers (collagen, thrombin, and U46619) were used to determine the antiplatelet effects of costunolide. Transmission aggregometry tracing showed that costunolide (20 and 50 µM) markedly inhibited collagen-induced platelet aggregation (Figure 1B). At a concentration of 50 µM, costunolide achieved approximately 90% inhibition (Figure 1E). However, at concentrations of 20–100 µM, costunolide had no effect on platelet aggregation induced by thrombin or U46619 (Figure 1C–E). These findings indicate that costunolide selectively suppresses collagen-mediated platelet activation.

### 3.2. Costunolide Blocks Collagen-Induced Granule Release

**Figure 2:**
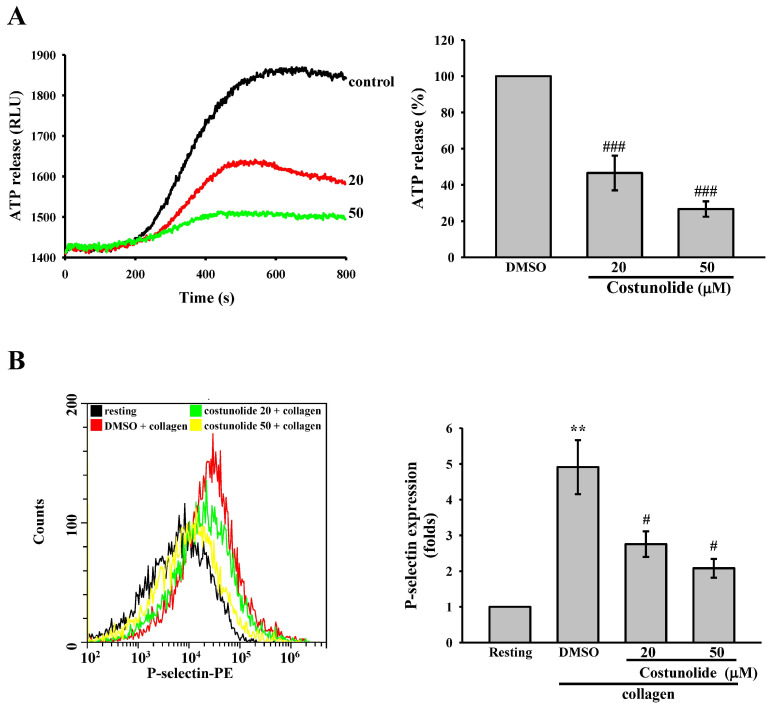
Costunolide inhibits the release of ATP and P-selectin stimulated by collagen. Washed platelets (3.6 × 10^8^ cells/mL) were pretreated with 0.1% DMSO (solvent control) or costunolide (20 and 50 µM) prior to the addition of 1 μg/mL collagen to trigger (**A**) ATP release and (**B**) P-selectin secretion. Luciferase/luciferin and phycoerythrin (PE)–P-selectin antibodies were used to determine ATP release and P-selectin expression, respectively. Data are presented as means ± standard errors of the mean (*n* = 4). (**A**) ^###^ *p* < 0.001, compared with the DMSO (solvent control) group. (**B**) ** *p* < 0.01, compared with the resting group. ^#^ *p* < 0.05, compared with the DMSO (solvent control) group.

During platelet activation, granule-derived molecules such as ADP (or ATP) stored at dense granule and P-selectin stored at alpha granule are released, and this contributes to platelet activation and circulating platelet recruitment [22]. Accordingly, we measured ATP release and P-selectin expression to evaluate the potential of costunolide in preventing granule release. ATP release was quantified using a luciferase–luciferin bioluminescence assay. The peak luminescence intensity was analyzed to evaluate the effects of costunolide on ATP release. The results indicated that collagen strongly triggered ATP release, as evidenced by a significant elevation in luminescence intensity, and this response was reversed by costunolide (20 and 50 µM) (Figure 2A). In addition, P-selectin expression was detected by antibodies against P-selectin conjugated with phycoerythrin through flow cytometry. The fold change in mean fluorescence intensity relative to resting groups was determined to evaluate the effect of costunolide on P-selectin expression. The data revealed that collagen significantly upregulated P-selectin expression on the surface of platelets, as evidenced by increased fluorescence intensity, which was attenuated by costunolide (20 and 50 µM) (Figure 2B). These findings indicate that costunolide can attenuate platelet activation by reducing the release of dense and alpha granules.

### 3.3. Costunolide Prevents Collagen-Induced Calcium Mobilization and GPIIb/IIIa Activation

**Figure 3:**
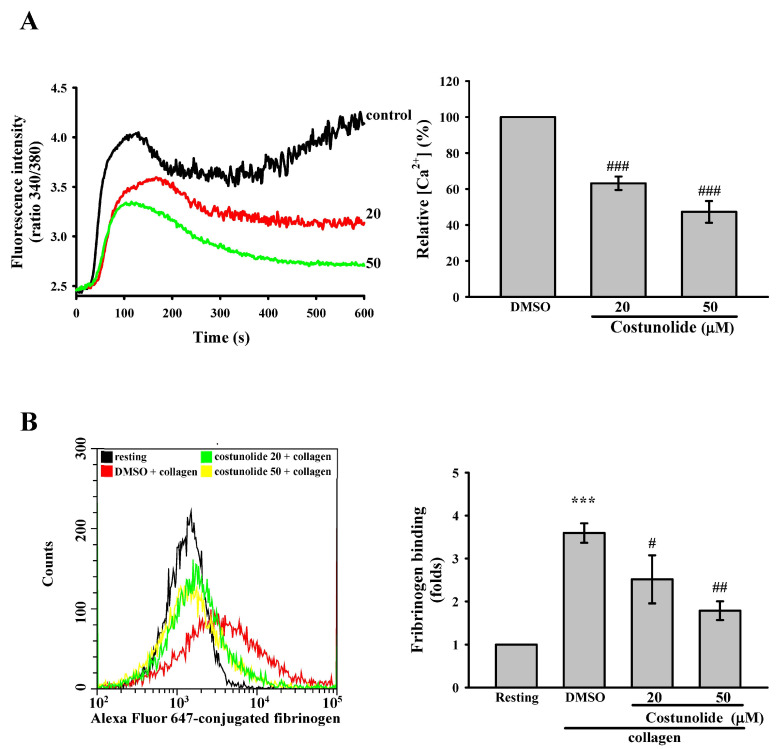
Costunolide inhibits collagen-induced calcium mobilization and GPIIb/IIIa activation. Washed platelets (3.6 × 10^8^ cells/mL) were preincubated with 0.1% DMSO (solvent control) or costunolide (20 and 50 µM) prior to the addition of 1 μg/mL collagen to induce (**A**) calcium mobilization and (**B**) GPIIb/IIIa activation. Fura 2-AM and Alexa Fluor 647 fibrinogen conjugate were used to detect changes in the levels of intracellular calcium and GPIIb/IIIa activation, respectively. Data are presented as means ± standard errors of the mean (*n* = 4). (**A**) ^###^ *p* < 0.001, compared with the DMSO (solvent control) group. (**B**) *** *p* < 0.001, compared with the resting group. ^#^ *p* < 0.05 and ^##^ *p* < 0.01, compared with the DMSO (solvent control) group.

An increase in cytosolic calcium in platelets is essential for activation processes such as shape change, degranulation, and inside-out activation of GPIIb/IIIa [23]. Therefore, we examined whether costunolide affects cytosolic calcium levels. Fura-2 AM was used to monitor cytosolic calcium dynamics; an increase in the ratio of fluorescence (340/380 nm) was considered to indicate an increase in intracellular calcium levels. The data revealed that collagen induced an increase in cytosolic calcium levels, which were reduced by costunolide (20 and 50 µM; Figure 3A). Additionally, GPIIb/IIIa can mediate platelet aggregation by binding fibrinogen, which forms bridges between adjacent platelets [24]. Platelet activation can change the conformation of GPIIb/IIIa into a functional receptor for fibrinogens [25]. Accordingly, the level of fibrinogen binding to GPIIb/IIIa was measured to evaluate the extent of GPIIb/IIIa activation. In this study, Alexa Fluor 647-conjugated fibrinogen was employed to quantify GPIIb/IIIa activation, which was assessed by calculating fold change in mean fluorescence intensity through flow cytometry to determine GPIIb/IIIa activation. The data indicated that collagen induced strong fibrinogen binding (red line, fluorescence intensity shifting to the right) relative to the resting group, which was reversed by costunolide (20 and 50 µM), suggesting that costunolide can inhibit collagen-induced GPIIb/IIIa activation (Figure 3B). Collectively, these findings indicate that costunolide may block collagen-stimulated platelet activation, partly through suppressing granule release, calcium mobilization, and GPIIb/IIIa activation.

### 3.4. Costunolide Prevents PLCγ2-PKC, Akt, and MAPK Signaling Pathways

**Figure 4:**
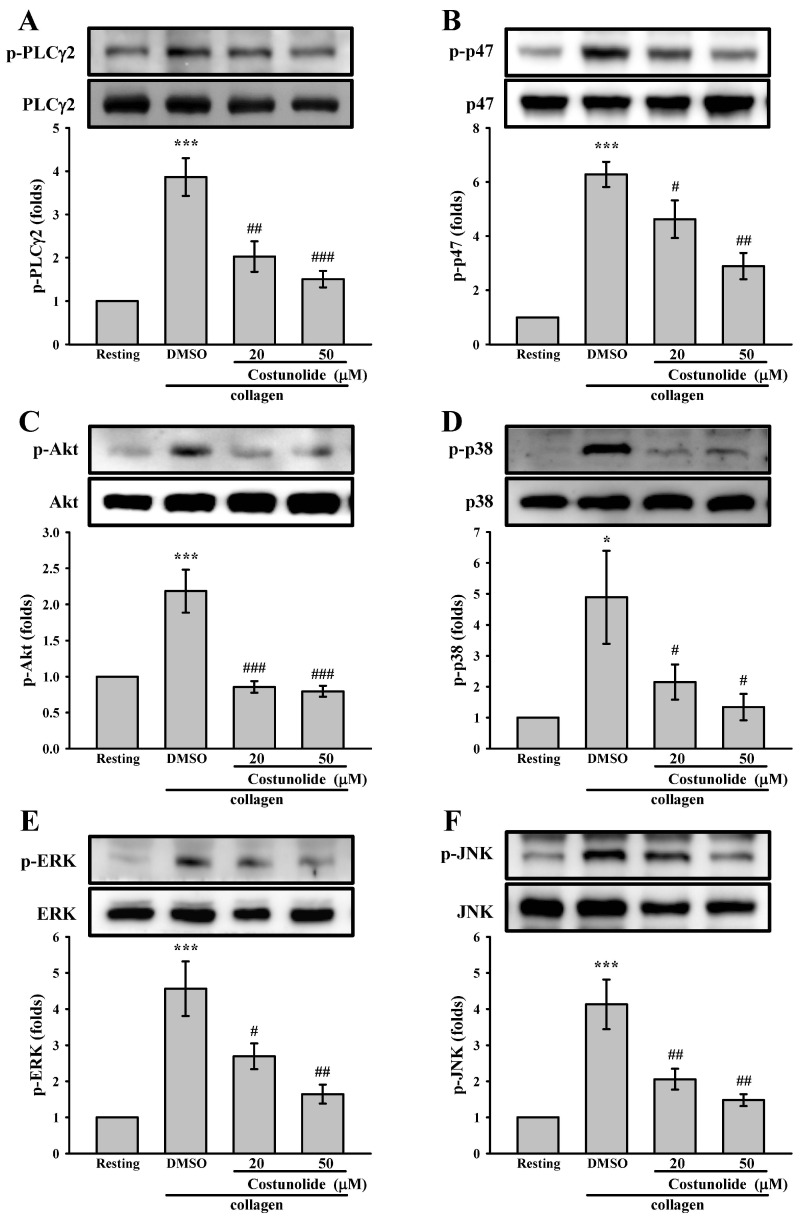
Costunolide reduces collagen-induced PLCγ2, p47, Akt, and MAPK phosphorylation. Washed platelets (3.6 × 10^8^ cells/mL) were pretreated with 0.1% DMSO (solvent control) or costunolide (20 and 50 µM) prior to the addition of 1 μg/mL collagen to trigger (**A**) PLCγ2, (**B**) p47, (**C**) Akt, (**D**) p38, (**E**) ERK and (**F**) JNK phosphorylation. Subcellular extracts were then subjected to Western blot analyses. Data are presented as means ± standard errors of the mean (*n* = 4). * *p* < 0.05 and *** *p* < 0.001, compared with the resting group. ^#^ *p* < 0.05, ^##^ *p* < 0.01, and ^###^ *p* < 0.001, compared with the DMSO (solvent control) group.

On the basis of the selectivity of costunolide in blocking collagen-stimulated platelet activation, GPVI downstream signaling pathways, including PLCγ2 and PKC, were determined. Our results indicated that collagen strongly induced the phosphorylation of PLCγ2 and p47 (PKC substrates), which was inhibited by costunolide (20 and 50 µM; Figure 4A,B). We further examined the effects of costunolide on Akt and MAPKs (p38 MAPK, ERK, and c-Jun N-terminal kinase), which are involved in collagen-stimulated platelet activation [26,27,28]. We observed that costunolide markedly reduced the collagen-induced levels of Akt and MAPK phosphorylation (Figure 4C–F). This finding suggested that costunolide inhibited collagen-stimulated platelet activation signaling. Collectively, these findings suggest that costunolide prevents granule release, calcium mobilization, and GPIIb/IIIa activation, partly through suppressing PLCγ2-PKC, Akt, and MAPK signaling pathways, and finally blocks collagen-stimulated platelet activation and aggregation.

### 3.5. Costunolide Inhibits Thrombus Formation Without Bleeding Tendency in Mice

**Figure 5:**
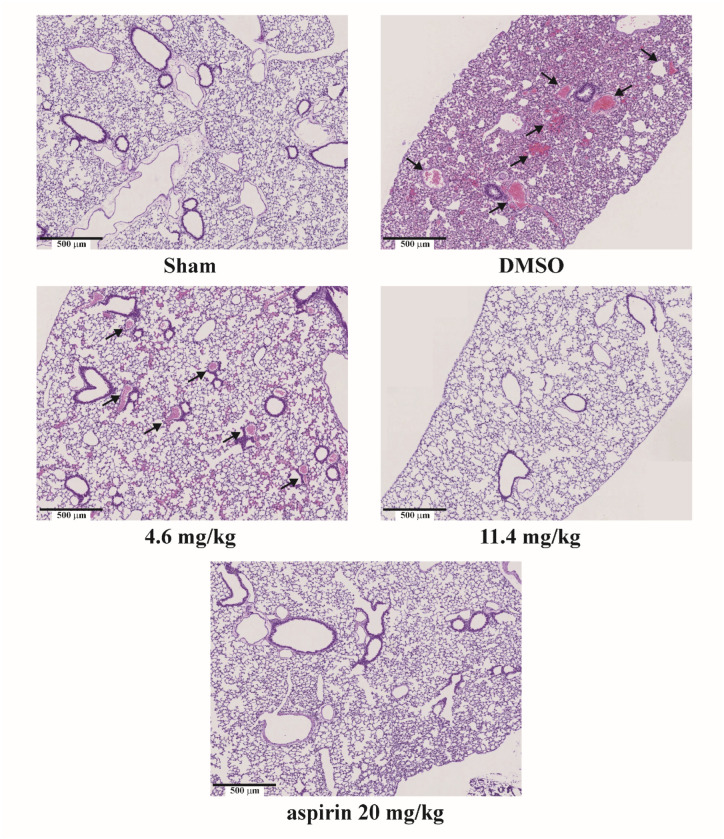

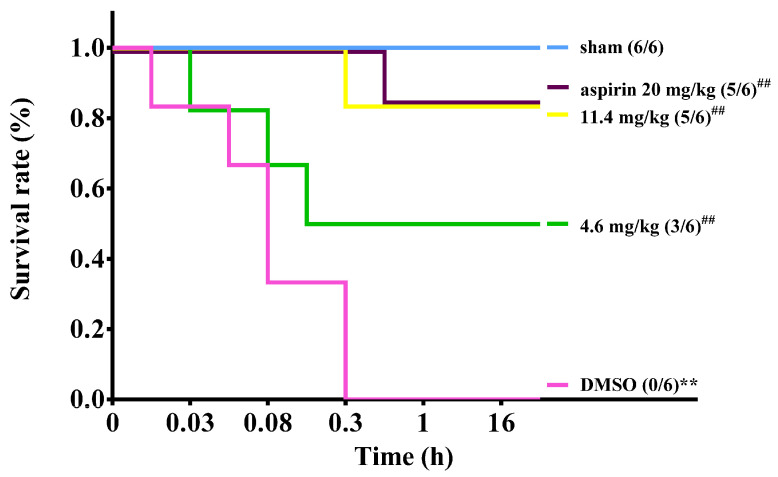
Costunolide inhibits collagen/epinephrine-induced pulmonary thromboembolism in mice. Mice were treated intravenously with 1.1 g/kg DMSO (solvent control), costunolide (4.6 and 11.4 mg/kg), or 20 mg/kg aspirin (positive control) for 10 min and then with collagen and epinephrine to trigger lung thrombosis. Excised lung tissues were subjected to hematoxylin and eosin staining for evaluating thrombus formation. Arrows indicate the presence of thrombi within the pulmonary vessels. Survival rate of mice was monitored within 24 h. Data are presented as means ± standard errors of the mean (*n* = 6). ** *p* < 0.01, compared with the sham group. ^##^ *p* < 0.01, compared with the DMSO (solvent control) group.

Our in vitro results suggested that costunolide has an antiplatelet effect. Therefore, we examined whether costunolide also has an antithrombotic effect. Collagen and epinephrine were used to trigger thrombus formation in the lungs of mice. At the end of the thrombosis experiment, sections of the lungs were stained with hematoxylin and eosin to observe whether thrombus formation had occurred. As shown in Figure 5, the results indicated that collagen/epinephrine markedly induced considerable pulmonary thrombosis (indicated by arrows in the DMSO group) and resulted in 100% mortality (6/6), which was observed for 24 h after their administration. In contrast, aspirin (20 mg/kg), as a positive control, markedly attenuated lung thrombosis and increased the survival rate (5/6 surviving mice). Similarly, costunolide (4.6 and 11.4 mg/kg) inhibited pulmonary thrombosis and increased the survival rate (3/6 and 5/6 surviving mice, respectively).

**Figure 6:**
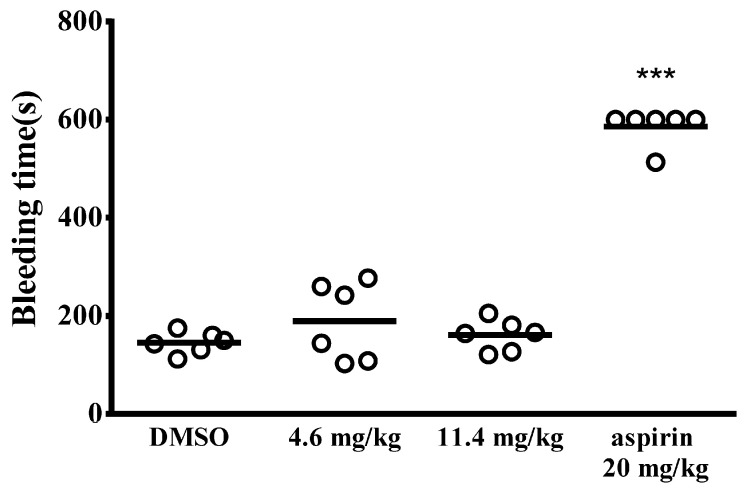
Costunolide does not affect hemostasis in vivo. Mice were treated intravenously with saline (control), 1.1 g/kg DMSO (solvent control), costunolide (4.6 and 11.4 mg/kg), or 20 mg/kg aspirin (positive control) for 10 min. To initiate bleeding, a tail transection was performed 3 mm from the tip, followed by immediate immersion in saline. The bleeding duration was monitored until it ceased for at least 10 s, with a maximum observation period of 10 min. Each point in the plot indicates a mouse. Data are presented as means ± standard errors of the mean (*n* = 6). *** *p* < 0.001, compared with the DMSO group.

In addition, mesenteric thrombus formation was induced by fluorescein sodium under ultraviolet irradiation. This process triggers endothelial damage, leading to subsequent vessel occlusion. As shown in Supplementary Figure S1, vessel occlusion (indicated by arrows) occurred 160 s after ultraviolet irradiation in the DMSO group. Both costunolide and aspirin markedly prolonged the occlusion time compared to the DMSO group. Notably, while aspirin markedly increased bleeding time, costunolide had no significant effect on bleeding (Figure 6). These findings suggest that costunolide exerts antithrombotic activity without an associated bleeding risk.

## 4. Discussion

This study explores the antiplatelet and antithrombotic effects of costunolide. It demonstrates that costunolide inhibits PLCγ2-PKC, Akt, and MAPK signaling pathways and subsequent granule release and GPIIb/IIIa activation, which in turn suppresses platelet activation, platelet aggregation, and thrombus formation.

Studies have indicated that costunolide exerts both anti-inflammatory and antioxidant effects, which may slow the progression of atherosclerosis. For example, costunolide was reported to alleviate ulcerative colitis by downregulating the expression of Toll-like receptor 4 (TLR4) [29], which is involved in the formation of atherosclerosis [30,31,32]. Several studies have suggested that costunolide exerts its anti-inflammatory effect by inhibiting NF-κB signaling in several disease cell models [10,13,33,34]. For example, costunolide was reported to inhibit the uptake of oxidized low-density lipoprotein in macrophages, leading to reduced expression of proinflammatory molecules and a reduction in atherosclerosis in high-fat-diet-fed ApoE^−/−^ mice by covalently binding to IκB kinase, an upstream regulator of NF-κB activation [15]. NF-κB is one of the key regulators of inflammation and contributes to the pathogenesis of atherosclerosis [35,36]. Notably, costunolide was demonstrated to covalently bind to cysteine 598 in the NACHT domain of NLRP3, inhibiting the assembly and activation of the NLRP3 inflammasome [37]. NLRP3 inflammasome reportedly plays a pivotal role in human atherosclerotic CVD [38,39]. These findings, including those regarding the effects of costunolide on TLR4, NF-κB, and NLRP3, highlight the therapeutic or preventive potential of costunolide against atherosclerotic CVD, particularly heart attack and stroke.

Despite the aforementioned findings, the antiplatelet effect of costunolide remains unclear. In this study, we examined the effect of costunolide on platelet activation. We found that costunolide inhibited the activation of PLCγ2, one key downstream signaling molecule of GPVI. PLCγ2 facilitates the formation of DAG and IP3, which respectively trigger PKC activation and calcium mobilization [22]. Costunolide also inhibits Akt activation, a signaling event downstream of GPVI [26,27]. A previous study has shown that the PI3K/Akt pathway regulates GPVI-mediated platelet aggregation, platelet secretion, and calcium mobilization [27]. Furthermore, it blunts the activation of MAPKs (p38, ERK, and JNK), which are essential for granule release, clot retraction, and GPIIb/IIIa activation [28,40,41,42]. Moreover, pharmacological inhibition or gene deletion of MAPKs significantly delayed thrombus formation [28]. These results suggest that costunolide disrupts the downstream signaling of GPVI. This blockade impairs granule release, calcium mobilization, and GPIIbIIIa activation, ultimately reducing platelet activation, platelet aggregation, and thrombus formation. Previously, platelets were reported to support the progression of atherosclerosis and mediate the thrombotic occlusion of ruptured or eroded plaques [43]. In addition, atherosclerotic plaque homogenates were demonstrated to induce platelet activation and thrombus formation, partly through GPVI signaling [44]. GPVI activation by fibrin has also been proposed to play a key feedback role in promoting coagulation and thrombin formation during thrombosis [45,46]. Accordingly, inhibiting platelet activation by blocking GPVI receptors or its downstream signaling may slow the progression of atherosclerosis and prevent thrombus formation due to ruptured or eroded plaques. Although we did not observe a direct effect of costunolide on GPVI in the present study (Supplementary Figure S2), we discovered that costunolide selectively reduced collagen-mediated platelet aggregation and blocked the downstream signaling of GPVI. Moreover, the present in vivo study suggested that costunolide prevented pulmonary thrombosis and thrombus formation in mesenteric vessels in mice without affecting hemostasis (Supplementary Figure S1). These findings outline the benefits of costunolide in cardiovascular disease, particularly stroke and heart attack.

In addition, the coagulation system is involved in hemostasis and thrombosis. Upon vascular injury, the tissue factor (TF)–factor VIIa complex initiates the coagulation cascade, leading to thrombin generation and fibrin formation. Thrombin subsequently activates platelets via PAR1 and PAR4, while fibrin or fibrin cross-linking stabilizes the hemostatic plug [47,48]. In pathological arterial thrombosis, the thrombus growth depends significantly on Factor XI’s ability to amplify thrombin generation [48]; therefore, targeting the coagulation pathway remains one of the major antithrombotic strategies. For example, statins exert beneficial pleiotropic effects independent of their cholesterol-lowering properties, such as reducing TF expression, thrombin generation, and the activity of several coagulation factors [49,50]. These effects may translate into clinical benefits; indeed, PE patients with pulmonary embolism using statins have been reported to have a significantly lower 30-day mortality risk compared to non-users [49]. A recent report also showed that costunolide significantly reduced elevated fibrinogen levels in the diabetic nephropathy model. However, whether costunolide can regulate the coagulation cascade and exert beneficial effects in venous thromboembolism remains to be elucidated.

In the present study, several limitations regarding costunolide remain to be addressed, particularly concerning drug concentrations and its molecular targets. For achieving a therapeutically effective plasma concentration, the structural modification of costunolide will be necessary to enhance its potency and achieve effective therapeutic concentrations. Furthermore, its precise molecular targets warrant further investigation.

## 5. Conclusions

In conclusion, besides its anti-inflammatory and antioxidative effects, costunolide exerts antiplatelet and antithrombotic effects, partly through suppressing PLCγ2–PKC, Akt, and MAPK signaling pathways, thereby inhibiting granule release and GPIIb/IIIa activation. In addition, costunolide may serve as a lead compound for the synthesis and discovery of novel derivatives—optimized through structural modification for enhanced antiplatelet efficacy—that could provide a promising therapeutic strategy for patients with CVD, particularly stroke and heart attack.

## Data Availability

The original contributions presented in this study are included in the article/Supplementary Materials. Further inquiries can be directed to the corresponding authors.

## Supplementary Materials

The following supporting information can be downloaded at https://www.mdpi.com/article/10.3390/cells15100938/s1, Figure S1: Costunolide prevents mesenteric thrombus formation in vivo; Figure S2: Costunolide does not affect convulxin-induced platelet aggregation.

## Abbreviations

ATP	adenosine triphosphate
CVDs	cardiovascular diseases
DMSO	dimethyl sulfoxide
ERK1/2	extracellular signal-regulated kinase 1/2
GPIIb/IIIa	glycoprotein IIb/IIIa
GPVI	glycoprotein VI
MAPK	mitogen-activated protein kinase
PLCγ2	phospholipase Cγ2

## References

[1] WHO (2025). Cardiovascular Diseases (CVDs). https://www.who.int/news-room/fact-sheets/detail/cardiovascular-diseases-(cvds).

[2] Davi G., Patrono C. (2007). Platelet activation and atherothrombosis. N. Engl. J. Med..

[3] Martinez Bravo G., Annarapu G., Carmona E., Nawarskas J., Clark R., Novelli E., Mota Alvidrez R.I. (2024). Platelets in Thrombosis and Atherosclerosis: A Double-Edged Sword. Am. J. Pathol..

[4] Massberg S., Brand K., Gruner S., Page S., Muller E., Muller I., Bergmeier W., Richter T., Lorenz M., Konrad I. (2002). A critical role of platelet adhesion in the initiation of atherosclerotic lesion formation. J. Exp. Med..

[5] Burger P.C., Wagner D.D. (2003). Platelet P-selectin facilitates atherosclerotic lesion development. Blood.

[6] Huo Y., Schober A., Forlow S.B., Smith D.F., Hyman M.C., Jung S., Littman D.R., Weber C., Ley K. (2003). Circulating activated platelets exacerbate atherosclerosis in mice deficient in apolipoprotein E. Nat. Med..

[7] Lim G.B. (2020). Pro-inflammatory atherogenic role of platelets. Nat. Rev. Cardiol..

[8] Borst O., Gawaz M. (2021). Glycoprotein VI—Novel target in antiplatelet medication. Pharmacol. Ther..

[9] Estevez B., Du X. (2017). New Concepts and Mechanisms of Platelet Activation Signaling. Physiology.

[10] Zhang S.Q., Deng Q., Tian C., Zhao H.H., Yang L.Y., Cheng X.W., Wang G.P., Liu D. (2025). Costunolide normalizes neuroinflammation and improves neurogenesis deficits in a mouse model of depression through inhibiting microglial Akt/mTOR/NF-κB pathway. Acta Pharmacol. Sin..

[11] Wang R., Zhao Y., Zhou L., Lin F., Wan M., Gan A., Wu B., Yan T., Jia Y. (2024). Costunolide ameliorates MNNG-induced chronic atrophic gastritis through inhibiting oxidative stress and DNA damage via activation of Nrf2. Phytomedicine.

[12] Jin B., Chen Y., Wang J., Chen Y., Zhang M., Huang J., Wang Y. (2023). Costunolide alleviates hyperglycaemia-induced diabetic cardiomyopathy via inhibiting inflammatory responses and oxidative stress. J. Cell. Mol. Med..

[13] Ye B., Chen X., Chen Y., Lin W., Xu D., Fang Z., Chattipakorn N., Huang W., Wang X., Wu G. (2023). Inhibition of TAK1/TAB2 complex formation by costunolide attenuates obesity cardiomyopathy via the NF-κB signaling pathway. Phytomedicine.

[14] Liu W., Yang W., Niu R., Cong L., Jiang M., Bai G. (2023). Costunolide covalently targets and inhibits CaMKII phosphorylation to reduce ischemia-associated brain damage. Phytomedicine.

[15] Huang Z.Q., Luo W., Li W.X., Chen P., Wang Z., Chen R.J., Wang Y., Huang W.J., Liang G. (2023). Costunolide alleviates atherosclerosis in high-fat diet-fed ApoE^−/−^ mice through covalently binding to IKKβ and inhibiting NF-κB-mediated inflammation. Acta Pharmacol. Sin..

[16] Huang H., Park S., Zhang H., Park S., Kwon W., Kim E., Zhang X., Jang S., Yoon D., Choi S.K. (2021). Targeting AKT with costunolide suppresses the growth of colorectal cancer cells and induces apoptosis in vitro and in vivo. J. Exp. Clin. Cancer Res..

[17] Jin X., Wang C., Wang L. (2020). Costunolide inhibits osteosarcoma growth and metastasis via suppressing STAT3 signal pathway. Biomed. Pharmacother..

[18] Lin K.H., Hsiao G., Shih C.M., Chou D.S., Sheu J.R. (2009). Mechanisms of resveratrol-induced platelet apoptosis. Cardiovasc. Res..

[19] Shih T.L., Lin K.H., Chen R.J., Chen T.Y., Kao W.T., Liu J.W., Wang H.H., Peng H.Y., Sun Y.Y., Lu W.J. (2021). A novel naphthalimide derivative reduces platelet activation and thrombus formation via suppressing GPVI. J. Cell. Mol. Med..

[20] National Research Council (US), Committee for the Update of the Guide for the Care and Use of Laboratory Animals (2011). Guide for the Care and Use of Laboratory Animals.

[21] Reagan-Shaw S., Nihal M., Ahmad N. (2008). Dose translation from animal to human studies revisited. FASEB J..

[22] Li Z., Delaney M.K., O’Brien K.A., Du X. (2010). Signaling during platelet adhesion and activation. Arter. Thromb. Vasc. Biol..

[23] Varga-Szabo D., Braun A., Nieswandt B. (2009). Calcium signaling in platelets. J. Thromb. Haemost..

[24] Nurden A.T., Poujol C., Durrieu-Jais C., Nurden P. (1999). Platelet glycoprotein IIb/IIIa inhibitors: Basic and clinical aspects. Arter. Thromb. Vasc. Biol..

[25] Sims P.J., Ginsberg M.H., Plow E.F., Shattil S.J. (1991). Effect of platelet activation on the conformation of the plasma membrane glycoprotein IIb-IIIa complex. J. Biol. Chem..

[26] Barry F.A., Gibbins J.M. (2002). Protein kinase B is regulated in platelets by the collagen receptor glycoprotein VI. J. Biol. Chem..

[27] Kim S., Mangin P., Dangelmaier C., Lillian R., Jackson S.P., Daniel J.L., Kunapuli S.P. (2009). Role of phosphoinositide 3-kinase β in glycoprotein VI-mediated Akt activation in platelets. J. Biol. Chem..

[28] Patel P., Naik U.P. (2020). Platelet MAPKs-a 20+ year history: What do we really know?. J. Thromb. Haemost..

[29] Liang S., Chu C., Li R., Jiang G., Du L. (2024). Costunolide and dehydrocostus lactone alleviate ulcerative colitis via regulating TLR4, NF-κB and PI3K expression. Sci. Rep..

[30] Fuster J.J. (2018). TLR4 in Atherogenesis: Paying the Toll for Antimicrobial Defense. J. Am. Coll. Cardiol..

[31] Singh R.K., Haka A.S., Asmal A., Barbosa-Lorenzi V.C., Grosheva I., Chin H.F., Xiong Y., Hla T., Maxfield F.R. (2020). TLR4 (Toll-like Receptor 4)-Dependent Signaling Drives Extracellular Catabolism of LDL (Low-Density Lipoprotein) Aggregates. Arter. Thromb. Vasc. Biol..

[32] Michelsen K.S., Wong M.H., Shah P.K., Zhang W., Yano J., Doherty T.M., Akira S., Rajavashisth T.B., Arditi M. (2004). Lack of Toll-like receptor 4 or myeloid differentiation factor 88 reduces atherosclerosis and alters plaque phenotype in mice deficient in apolipoprotein E. Proc. Natl. Acad. Sci. USA.

[33] Han T., Tang H., Lin C., Yan D., Zhou Z., Yang Y., Cai L., Zhu J., Gao B., Si Y. (2024). Costunolide mitigates inflammation and promotes extracellualr matrix integrity of thoracic aortic dissection by inhibiting NF-κB signaling. Int. Immunopharmacol..

[34] Zhao Y., Wang Y.H., Tu W.C., Wang D.W., Lu M.J., Shao Y. (2024). Costunolide Inhibits Chronic Kidney Disease Development by Attenuating IKKβ/NF-κB Pathway. Drug Des. Dev. Ther..

[35] de Winther M.P., Kanters E., Kraal G., Hofker M.H. (2005). Nuclear factor κB signaling in atherogenesis. Arter. Thromb. Vasc. Biol..

[36] Gareus R., Kotsaki E., Xanthoulea S., van der Made I., Gijbels M.J., Kardakaris R., Polykratis A., Kollias G., de Winther M.P., Pasparakis M. (2008). Endothelial cell-specific NF-κB inhibition protects mice from atherosclerosis. Cell Metab..

[37] Xu H., Chen J., Chen P., Li W., Shao J., Hong S., Wang Y., Chen L., Luo W., Liang G. (2023). Costunolide covalently targets NACHT domain of NLRP3 to inhibit inflammasome activation and alleviate NLRP3-driven inflammatory diseases. Acta Pharm. Sin. B.

[38] Tall A.R., Bornfeldt K.E. (2023). Inflammasomes and Atherosclerosis: A Mixed Picture. Circ. Res..

[39] Delfos L., Depuydt M.A.C., Chemaly M., Coyle S., Schaftenaar F.H., van Santbrink P.J., Lindenbergh P.P., Bernabe Kleijn M.N.A., Costello C., Power C.A. (2025). NLRP3 Inflammasome Inhibition by the Novel Bispecific Antibody InflamAb Attenuates Atherosclerosis in Apolipoprotein E-Deficient Mice. JACC Basic Transl. Sci..

[40] Adam F., Kauskot A., Nurden P., Sulpice E., Hoylaerts M.F., Davis R.J., Rosa J.P., Bryckaert M. (2010). Platelet JNK1 is involved in secretion and thrombus formation. Blood.

[41] Flevaris P., Li Z., Zhang G., Zheng Y., Liu J., Du X. (2009). Two distinct roles of mitogen-activated protein kinases in platelets and a novel Rac1-MAPK-dependent integrin outside-in retractile signaling pathway. Blood.

[42] Li Z., Xi X., Du X. (2001). A mitogen-activated protein kinase-dependent signaling pathway in the activation of platelet integrin α_IIb_β_3_. J. Biol. Chem..

[43] Coenen D.M., Heinzmann A.C.A., Karel M.F.A., Cosemans J., Koenen R.R. (2021). The multifaceted contribution of platelets in the emergence and aftermath of acute cardiovascular events. Atherosclerosis.

[44] Jooss N.J., Smith C.W., Slater A., Montague S.J., Di Y., O’Shea C., Thomas M.R., Henskens Y.M.C., Heemskerk J.W.M., Watson S.P. (2022). Anti-GPVI nanobody blocks collagen- and atherosclerotic plaque-induced GPVI clustering, signaling, and thrombus formation. J. Thromb. Haemost..

[45] Alshehri O.M., Hughes C.E., Montague S., Watson S.K., Frampton J., Bender M., Watson S.P. (2015). Fibrin activates GPVI in human and mouse platelets. Blood.

[46] Mammadova-Bach E., Ollivier V., Loyau S., Schaff M., Dumont B., Favier R., Freyburger G., Latger-Cannard V., Nieswandt B., Gachet C. (2015). Platelet glycoprotein VI binds to polymerized fibrin and promotes thrombin generation. Blood.

[47] Li X., Sim M.M.S., Wood J.P. (2020). Recent Insights Into the Regulation of Coagulation and Thrombosis. Arter. Thromb. Vasc. Biol..

[48] Vilahur G., Fuster V. (2025). Interplay between platelets and coagulation: From protective haemostasis to pathological arterial thrombosis. Eur. Heart J..

[49] Siniscalchi C., Muriel A., Surinach Caralt J.M., Bikdeli B., Jimenez D., Lobo J.L., Amado C., Gil-Diaz A., Imbalzano E., Monreal M. (2022). Statin use and 30-day mortality in patients with acute symptomatic pulmonary embolism. J. Thromb. Haemost..

[50] Siniscalchi C., Basaglia M., Riva M., Meschi M., Meschi T., Castaldo G., Di Micco P. (2023). Statins Effects on Blood Clotting: A Review. Cells.

