# Supplementary material for: Costunolide, a Sesquiterpene Lactone, Protects Against Platelet Activation and Thrombus Formation
Source: Cells. 2026 May 20;15(10):938. doi: 10.3390/cells15100938 (PMC13204036; doi:10.3390/cells15100938)
Supplement: Supplementary file 1 [file cells-15-00938-s001.zip › cells-4285261-supplementary.pdf]

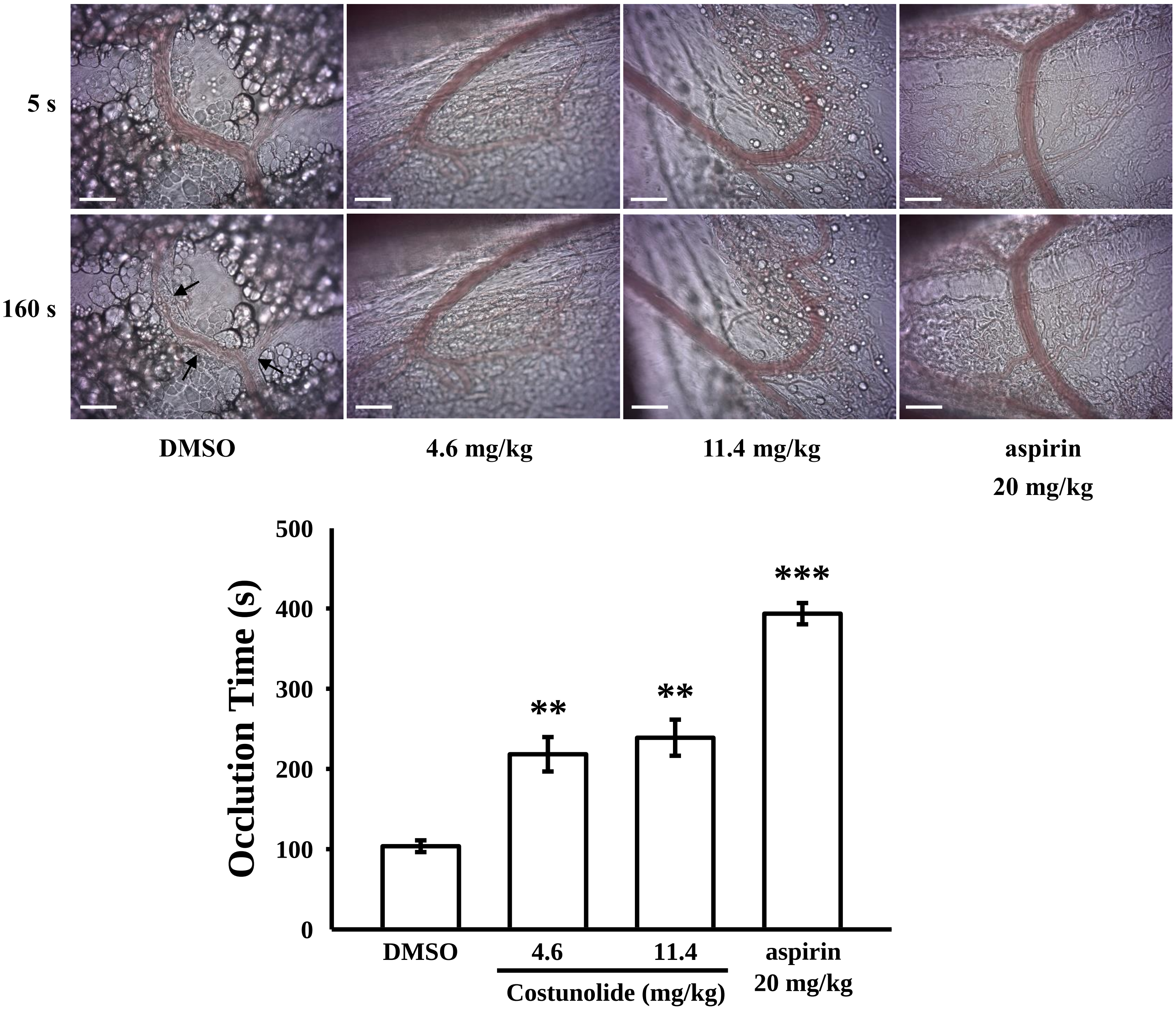

Supplementary Figure S1. Costunolide prevents mesenteric thrombus formation *in vivo*. Mice were treated intravenously with DMSO (1.1 g/kg, solvent control), costunolide (4.6 and 11.4 mg/kg), or aspirin (20 mg/kg, positive control) for 10 min. The endothelium of the mesenteric venules (30–40 μm) was damaged by ultraviolet irradiation, leading to vessel occlusion via thrombus formation (arrow). Scale bar = 50 μm. Data are presented as means ± standard errors of the mean (n = 3). $^{**}P$ < 0.01 and $^{***}P$ < 0.001, compared with the DMSO (solvent control) group.

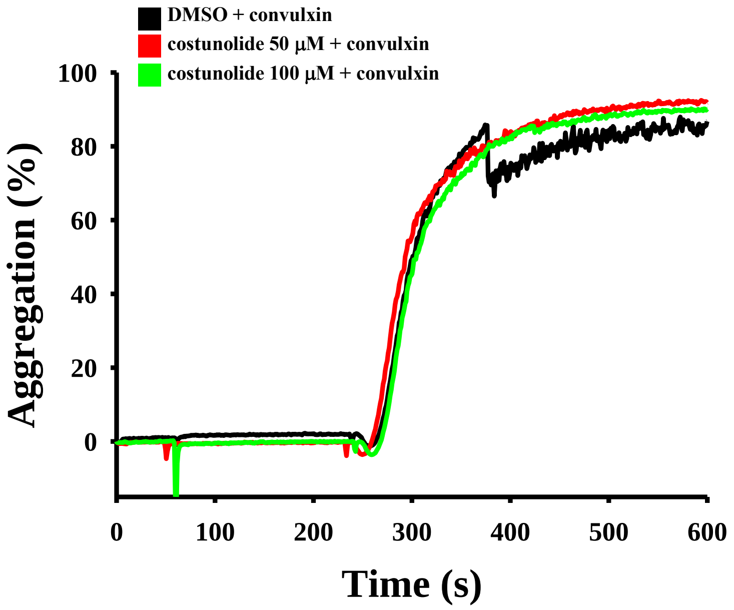

Supplementary Figure S2. Costunolide does not affect convulxin-induced platelet aggregation. Washed platelets (3.6×10^8 cells/mL) were pretreated with DMSO (0.1%, solvent control) or costunolide (50–100 μM) prior to the addition of with convulxin (10 ng/ml) to trigger platelet aggregation.
